# Supplementary material for: Empirical methods for controlling false positives and estimating confidence in ChIP-Seq peaks
Source: BMC Bioinformatics. 2008 Dec 5;9:523. doi: 10.1186/1471-2105-9-523 (PMC2628906; doi:10.1186/1471-2105-9-523)
Supplement: Additional File 1 — A variety of html documents from the USeq web site detailing the available applications, their best usage, output file type descriptions, command line menus, etc. [file 1471-2105-9-523-S1.zip › USeqUserGuides/JavaDoc.zip!/JavaDoc/index-files/index-21.html]

V-Index


|  |  |  |
| --- | --- | --- |
| **Overview** \| Package \| Class \| Use \| **Tree** \| **Deprecated** \| **Index** \| **Help** | |  |
| **PREV LETTER**   **NEXT LETTER** | **FRAMES**    **NO FRAMES**     **All Classes** |  |


A B C D E F G H I J L M N O P Q R S T U V W X Z 

---


## **V**

**value** - Variable in class trans.cel.Quantile: **VirtualCel** - Class in trans.graphics: Builds virtual chips from float[][] files and saves each as a PNG image file. **VirtualCel(String[])** - Constructor for class trans.graphics.VirtualCel: For generating png files. **VirtualCel(float[][], CelMasker)** - Constructor for class trans.graphics.VirtualCel: For integration with CelMasker. **VirtualCel(float[][], File, int)** - Constructor for class trans.graphics.VirtualCel: To write a matrix to png **VirtualCelFrame** - Class in trans.graphics: Frame for `VirtualCel`. **VirtualCelFrame()** - Constructor for class trans.graphics.VirtualCelFrame: **VirtualCelFrame(float[][], int, float)** - Constructor for class trans.graphics.VirtualCelFrame: **VirtualCelPanel** - Class in trans.graphics: Panel and calcs for `VirtualCel`. **VirtualCelPanel(float[][], int, float, VirtualCelFrame)** - Constructor for class trans.graphics.VirtualCelPanel: **VirtualCelPanel(float[][], int, File)** - Constructor for class trans.graphics.VirtualCelPanel: For printing pngs **VisibilityBean** - Class in cgar.beans: VisibilityBean: who can see it: Owner (person who entered the analysis), Group Members, WWW. **VisibilityBean()** - Constructor for class cgar.beans.VisibilityBean

---


|  |  |  |
| --- | --- | --- |
| **Overview** \| Package \| Class \| Use \| **Tree** \| **Deprecated** \| **Index** \| **Help** | |  |
| **PREV LETTER**   **NEXT LETTER** | **FRAMES**    **NO FRAMES**     **All Classes** |  |


A B C D E F G H I J L M N O P Q R S T U V W X Z 

---
